# Induction of Chemoresistance by All-Trans Retinoic Acid via a Noncanonical Signaling in Multiple Myeloma Cells

**DOI:** 10.1371/journal.pone.0085571

**Published:** 2014-01-09

**Authors:** Zhiqiang Liu, Tao Li, Kesheng Jiang, Qiaoli Huang, Yicheng Chen, Feng Qian

**Affiliations:** 1 Department of Lymphoma and Myeloma, Division of Cancer Medicine, Center for Cancer Immunology Research, the University of Texas MD Anderson Cancer Center, Houston, Texas, United States of America; 2 Department of Biology, College of Chemistry and Life Sciences, Zhejiang Normal University, Jinhua, Zhejiang, China; 3 Department of Urology, Sir Run Run Shaw Hospital, School of Medicine, Zhejiang University, Hangzhou, Zhejiang, China; 4 Department of Medical Function, Medical School of Yangtze University, Jingzhou, Hubei, China; New York University, United States of America

## Abstract

Despite the successful application of all-trans retinoic acid (ATRA) in multiple myeloma treatment, ATRA-induced chemoresistance in the myeloma patients is very common in clinic. In this study, we evaluated the effect of ATRA on the expression of apurinic endonuclease/redox factor-1 (Ape/Ref-1) in the U266 and RPMI-8226 myeloma cells to explore the chemoresistance mechanism involved. ATRA treatment induced upregulation of Ape/Ref-1 via a noncanonical signaling pathway, leading to enhanced pro-survival activity counteracting melphalan (an alkylating agent). ATRA rapidly activated p38-MSK (mitogen- and stress activated protein kinase) cascade to phosphorylate cAMP response element-binding protein (CREB). Phosphorylated CREB was recruited to the *Ape/Ref-1* promoter to evoke the gene expression. The stimulation of ATRA on Ape/Ref-1 expression was attenuated by either p38-MSK inhibitors or overexpression of dominant-negative MSK1 mutants. Moreover, ATRA-mediated Ape/Ref-1 upregulation was correlated with histone modification and activation of CBP/p300, an important cofactors for CREB transcriptional activity. C646, a competitive CBP/p300 inhibitor, abolished the upregulation of Ape/Ref-1 induced by ATRA. Intriguingly, CBP rather than p300 played a dominant role in the expression of Ape/Ref-1. Hence, our study suggests the existence of a noncanonical mechanism involving p38-MSK-CREB cascade and CBP induction to mediate ATRA-induced Ape/Ref-1 expression and acquired chemoresistance in myeloma cells.

## Introduction

All-trans retinoic acid (ATRA) is an active metabolite of vitamin A (retinol) and regulates a variety of important processes including development, differentiation, proliferation, and apoptosis in retinoic acid receptor (RAR) dependent canonical manner or noncanonical manners. The potent cyto-differentiating, pro-apoptotic, and growth-suppressive effects of ATRA have led to its application in the treatment of several malignant tumors [1]. There have been many studies regarding the potential therapeutic interest of ATRA in the multiple myeloma [2]. ATRA has been shown to inhibit the growth of myeloma cells by downregulation of the IL-6/IL-6R auto/paracrine loop, and upregulation of p21/Cip1 cyclin dependent kinase inhibitor [3], [4]. Recent study indicated that ATRA can downregulate total β-catenin and CD44 in myeloma cells, thereby impeding the proliferation and migration mediated by Wnt/β-catenin cascade [5]. ATRA also induced a decrease in Bcl-2 anti-apoptotic protein and an augment of Fas antigen, both facilitating progress along the apoptotic pathway [6]. Moreover, ATRA alone or combined with other anticancer agents can evoke significant differentiation in myeloma cells in parallel with the inhibition of tumor malignancy, restoring the gene expression and morphological characteristics of mature myeloma cells [7]–[11].

Despite clinical benefits of ATRA, the high incidence of intrinsic or acquired resistance to reduce ATRA responsiveness or cytotoxicity, has limited the application in cancer therapy. Pharmacokinetic studies have demonstrated that sustained ATRA administration induced a metabolic response consistent with a decline in plasma ATRA levels and total ATRA bioavailability, which were attributed to the induced expression of CYP26, a P450 enzyme mediating the conversion of ATRA [12]. ATRA-mediated functional modulation of Zyxin and PTOV1 would antagonize the activities of RARs to inhibit ATRA sensitivity [13]. Depending on the cellular context and differences in ATRA dosage and exposure periods, ATRA may induce the expression of several anti-apoptotic proteins, such as PKCδVIII, Bcl-2A1, cIAP2, Beclin1, and MDR1 (the multidrug resistance 1) [14], [15]. For example, ATRA alone or synergistic with a histone deacetylase inhibitor (HDAC1) depsipeptide can induce MDR1 expression and acquire multidrug-resistance in malignant cells [16]–[20]. The *MDR1* gene product P-gp functions as a trans-membrane efflux pump for diverse chemotherapeutic drugs.

Notably, apurinic endonuclease/redox factor-1 (Ape/Ref-1) is an important regulator implicated in the acquisition of MDR1-mediated multidrug resistance. Acetylated Ape/Ref-1 interacts with Y-box-binding protein 1 (YB-1) and enhances its binding to the Y-box element, leading to the transactivation of *MDR* [21], [22]. Ape/Ref-1 as an apurinic/apyrimidinic endonuclease in the DNA base excision repair (BER) pathway, accounts for repairing DNA damage induced by oxidative stress, alkylating agents, and ionizing radiation. Moreover, several transcription factors (including p53, NF-κB, HIF-1α, Egr-1, AP-1, and others) involved in tumor development and progression were revealed to be activated by Ape/Ref-1 via its redox activity. Accumulating studies have linked it to malignant phenotype and poor prognosis in several tumors, in particular to the sensitivity to radiotherapy and chemotherapy [23], [24].

In this study, we provided novel evidence that ATRA treatment upregulated Ape/Ref-1 expression in myeloma cells via a noncanonical signaling. Inhibition of p38-MSK cascade effectively abolished Ape/Ref-1 augmentation by ATRA. CREB phosphorylated by MSK and CBP was involved in ATRA-mediated Ape/Ref-1 upregulation. Herein, our findings elucidate a novel role of Ape/Ref-1 in ATRA-mediated chemoresistance in myeloma cells.

## Materials and Methods

### Reagents, Antibodies and Plasmids

ATRA, melphalan, SB203580, H89, C646, and *N*-acetyl L-cysteine (NAC) were purchased from Sigma-Aldrich (St. Louis, MO) and dissolved in dimethyl sulfoxide (DMSO). Aliquots were used only once and diluted in 1% FBS culture medium in the dark. FITC-conjugated Annexin V and propidium iodide (PI) were purchased from eBioscience (San Diego, CA).

Antibodies against Ape/Ref-1, GAPDH, CREB, MSK1, p300, and CBP were purchased from Santa Cruz Biotechnology (Santa Cruz, CA). Antibodies specific for p38, phospho-p38, phospho-MSK1 (Thr581), phospho-CREB (Ser133), histone H3, phospho-histone H3 (Ser10), acetyl-histone H3 (Lys9), and PARP-1 were supplied by Cell Signaling Technology (Danvers, MA). The primary antibody against MDR1 (ABCB1) was provided by Sigma.

siRNA vector against Ape/Ref-1 (pSUPER Ape/Ref-1) and negative control against firely luciferase (pSUPER Luc) were kind gifts from Dr. Demple B (Harvard School of Public Health, Boston, USA) [25]. The targeting sequence for *Ape/Ref-1* gene was 5′-GTCTGGTACGACTGGAGTA-3′. Mammalian expression vectors encoding wild-type (WT) and kinase-defective (KD) p38α (p38K53A) proteins were generous gift from Dr. Engelberg D (The Hebrew University of Jerusalem, Jerusalem, Israel) [26]. Plasmids expressing wild-type MSK1 and C-terminal kinase dead (CKD) were kindly provided by Dr. Bourgeade MF (Institut National de la Sante et de la Recherche Medicale, Paris, France). p300 and CBP expression vectors were kindly provided by Dr. Kouzarides T (University of Cambridge, Cambridge, UK). The luciferase reporters CREB-luc and pRSV-luc were derived from Stratagene and Promega companies, respectively. pcDNA3.1/β-gal vector was used as a control.

### Cell Culture and Transfection

Myeloma cell lines RPMI8226 and U266 have been described previously [27]. All myeloma cells were cultured in RPMI 1640 medium (Mediatech Cellgro, VA) supplemented with 10% fetal bovine serum (FBS) and antibiotics. Cells were cultured at 37°C in a humidified 5% CO_2_ atmosphere. Triplicate samples at a cell density of 0.2∼1×10^6^/ml were cultured with or without the desired concentrations of ATRA or other inhibitors added daily to the culture medium.

For transient transfections of plasmids, the Neon transfection system (Invitrogen) was used. Briefly, 1×10^6^ myeloma cells were mixed with 10 µg of plasmids and resuspended in 100 µl of resuspension buffer R, and the electroporation was performed under the condition of 1600 V, 20 Ω, and 1 pulse. The program was optimized for a successful gene transfer in >70% cells using a GFP plasmid, according to the manufacturer’s instructions. Transfected cells were then diluted at a density of 2×10^5^/ml and treated as described in the figure legends.

### Flow Cytometry

After treatments, myeloma cells were washed in 1×PBS and incubated at room temperature for 20 minutes with the FITC-conjugated Annexin V antibody. After incubation and washing, samples were analyzed on a LSR Fortessa (Becton Dickinson), and data were analyzed with FlowJo software.

### Cell Proliferation Assay

Cells were treated with ATRA at appropriate concentrations as indicated and cell proliferation was determined at 72 h post treatment using the Cell Counting Kit-8 (CCK-8) according to the manufacturer’s protocol (Dojindo). Absorbance was measured at 450 nm using a microplate reader (Bio-Rad). The values were used to calculate cell amount by setting the normal control as 100%.

### Western Blotting

Cells were harvested and lysed with lysis buffer (Cell Signaling Technology, MA). Cell lysates were subjected to SDS-PAGE, transferred to a Nitrocellulose membrane, and immunoblotted with antibodies. The membrane was stripped and re-immunoblotted with anti-GAPDH antibody to ensure equal protein loading. Secondary antibodies conjugated to horseradish peroxidase were used for detection, followed by enhanced chemiluminescence (Pierce Biotechnology, IL). Band intensity from immunoblotting images was quantified by Multi Gauge V3.0 (Fujifilm, Japan). The figures shown are representative of at least three independent experiments.

### RNA Interference (RNAi)

U266 cells (1×10^6^) were transfected either with 20 nM of a scrambled control siRNA or a specific siRNA that targets MSK1 (sc-35977, Santa Cruz) in Opti-MEM medium (Invitrogen) according to the transfection program described as above.

### Luciferase Assays

U266 cells were transfected with either non-target control or *MSK1* siRNA together with CREB-luc reporter and pRSV-luc plasmid, and cultured for 24 h. After ATRA treatment for another 24 h, luciferase activity was measured and normalized to *Renilla* luciferase activity. All experiments were done in triplicates and performed at least three times.

### RT-PCR and Real-time PCR

Total RNA was isolated using an RNeasy kit (Qiagen). The total RNA (1 µg) was subjected to reverse transcription using a SuperScript II (Invitrogen) reverse transcriptase PCR kit. From the resulting cDNA, 1 µl was amplified by PCR using 2.5 U of *Taq* DNA polymerase (Invitrogen) for 25–30 cycles. The primers utilized for RT-PCR include: *PP2Acα* 5′-AATCCAACGTGCAAGAGGT-3′ (forward), 5′-TCCAAACATTTGCATTTCCA-3′ (reverse); *PP1cα* 5′- ATGCTGGGGGGGGGTCAC-3′ (forward), 5′-CCTTTATTCAAGAGACCAGATGGG-3′ (reverse); *GAPDH* 5′-CCCCTTCATTGACCTCAACT-3′ (forward), 5′-TTGTCATGGATGACCTTGGC-3′ (reverse). 1 µl of the final cDNA was applied to real-time PCR amplification with SYBR-Green using a StepOnePlus real-time PCR system (Applied Biosciences). For real-time PCR, the primers include: *MDR1* 5′-AGACATGACCAGGTATGCCTAT-3′ (forward), 5′-AGCCTATCTCCTGTCGCATTA-3′ (reverse); *GAPDH* 5′-CAGCCTCAAGATCATCAGCA-3′ (forward), 5′-TGTGGTCATGAGTCCTTCCA-3′ (reverse); *Ape/Ref-1* 5′-GCTCTTGGAATGTGGATGGG-3′ (forward), 5′-GGAGCTGACCAGTATTGATGAGA-3′ (reverse) (data not shown). The fold change in the target gene relative to the *GAPDH* endogenous control gene is determined by: Fold Change = 2^−Δ(ΔCT)^, where ΔC_T_ = C_T(target)_ – C_T(GAPDH)_, and Δ(ΔC_T_) = ΔC_T(treated)_ − ΔC_T(control)_. Each value presents the average of at least 3 independent experiments.

### Chromatin Immunoprecipitation (ChIP) Assay

Chromatin immunoprecipitation assay ChIP was performed as previously described [28]. Briefly, after cross-linking with 1% formaldehyde, U266 cells were lysed and sonicated. Prior to immunoprecipitation with antibodies against phosphorylated CREB, acetyl- and phospho-histone H3, a small aliquot of chromatin was saved and used as an input control. The primers used for the *Ape/Ref-1* promoter were 5′-CACGTCCAAGTGCCTGTAAC-3′ (forward) and 5′-GGTGTCTTTCCCAGTGCTATCT-3′ (reverse).

### Statistical Analysis

All data in this study were displayed as means ± SD if not otherwise indicated. Comparisons were analyzed by Student’s *t*-test or one way ANOVA. The significance was analyzed with SPSS10.0 software and a *P*-value <0.05 was considered statistically significant. All results were reproduced in at least three independent experiments.

## Results

### ATRA Upregulates Ape/Ref-1 Protein in U266 and RPMI8226 Myeloma Cells

**Figure 1:**
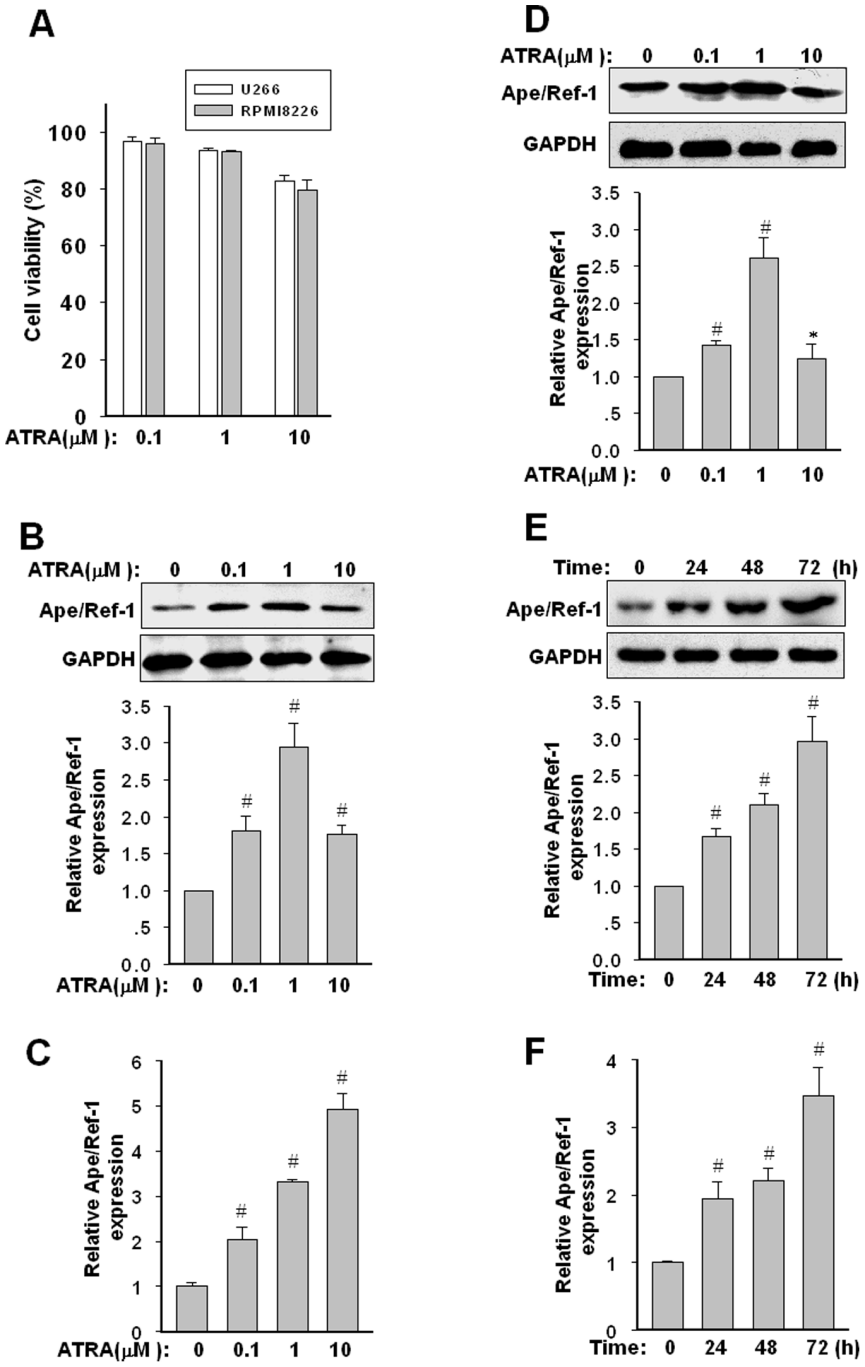
Effect of ATRA on the expression of Ape/Ref-1 in U266 and RPMI8226 myeloma cells. (**A**) Cytotoxic effects of ATRA in U266 and RPMI8226 cells were defined by flow cytometry analysis. The percentage of survival cells in each group was calculated from flow cytometry data and shown in histogram. Graphs show the mean ± SD of results from three independent experiments. (**B**) Expression of Ape/Ref-1 protein in U266 cells exposure to increasing dosage of ATRA. Myeloma cells were treated with the indicated concentrations of ATRA for 72 h. The cell lysates were analyzed for Ape/Ref-1 and GAPDH proteins by Western blotting. The ratio of Ape/Ref-1 to GAPDH was calculated by band-intensity analysis and expressed relative to that of untreated U266 cells. The values are means ± SD of three independent experiments. (**C**) mRNA level of *Ape/Ref-1* was measured by real-time PCR and normalized to *GAPDH* mRNA level. (**D**) Expression of Ape/Ref-1 protein in RPMI8226 cells exposure to increasing dosage of ATRA. (**E and F**) Time course of induced upregulation in Ape/Ref-1 protein and mRNA by 1 µM ATRA in U266 cells. All results are expressed as mean ± SD from three independent experiments. *P<0.05, ^#^ *P*<0.01, versus the control group untreated by ATRA.

Multiple myeloma cells may undergo growth inhibition, differentiation or apoptosis upon exposure to ATRA. After exposure to increasing concentrations of ATRA ranging from 0.1 to 10 µM for 72 h, the cytotoxic effects of ATRA on U266 and RPMI8226 cells were evaluated by flow cytometry analysis. As shown in **Fig. 1A**, 0.1 and 1 µM of ATRA showed no significant pro-apoptotic effect, whereas the concentration of ATRA exceeding 10 µM induced an obvious toxicity. Subsequently, effect of ATRA on the expression of Ape/Ref-1 protein was analyzed. As shown in **Fig. 1B**, ATRA up to 1 µM induced an elevated expression of Ape/Ref-1 level (about 2.9±0.3 fold) in a dose-dependent manner in U266 cells. However, high dosage of ATRA did not induce upregulation of the Ape/Ref-1 protein. Paradoxically, the mRNA level of *Ape/Ref-1* still exhibited an increasing elevation after exposure to ATRA at 10 µM (**Fig. 1C**). Similar results can be observed in RPMI8226 cells (**Fig. 1D**, mRNA data not shown). Meanwhile, the protein and mRNA levels of Ape/Ref-1 in U266 cells were also upregulated gradually by 1 µM ATRA treatment in a time-dependent manner (**Fig. 1E and F**).

### Ape/Ref-1 Inhibition Enhances the Cytotoxic Effect of ATRA

**Figure 2:**
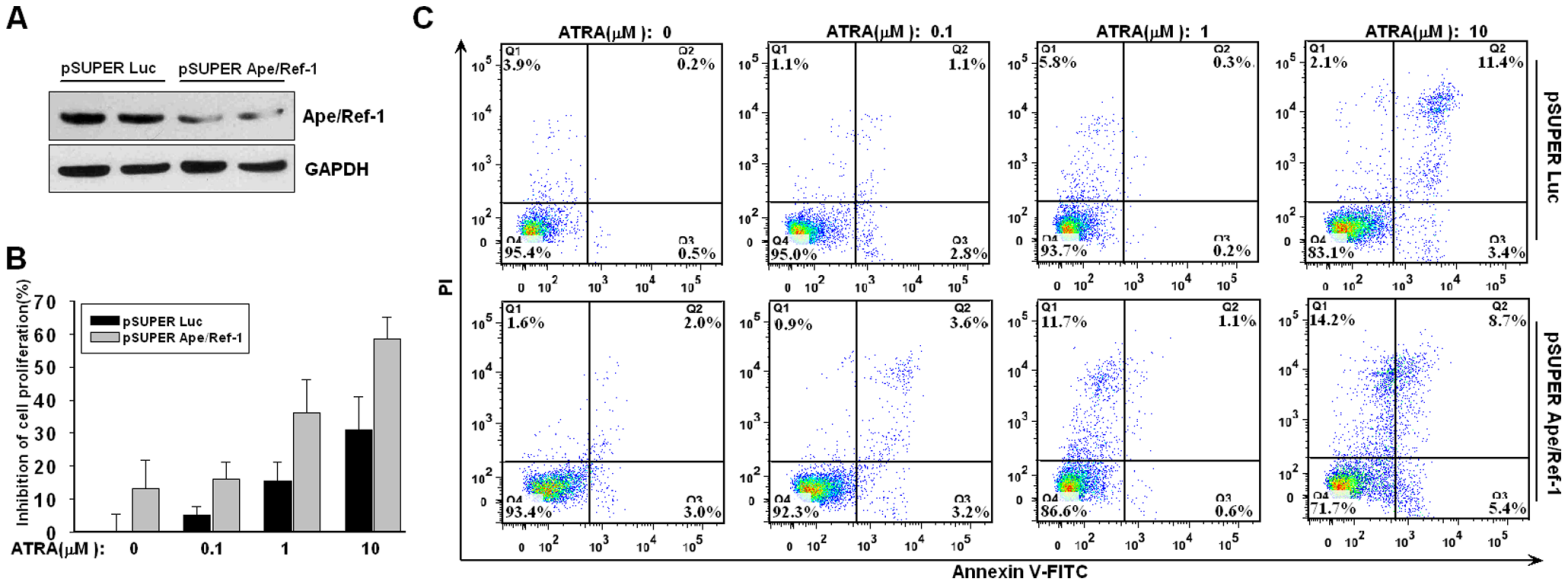
Ape/Ref-1 inhibition accelerates ATRA-induced growth arrest and apoptosis. (**A**) U266 cells were transfected with specific siRNA vector (pSUPER Aper/Ref-1) or control vector (pSUPER Luc), and incubated for 48 h following by immunoblotting with antibodies againset Ape/Ref-1 or GAPDH. (**B**) After transfected with siRNA or control vectors for 24 h, U266 cells were treated with the indicated concentrations of ATRA for 72 h and the proliferation rates were monitored by CCK-8 assay. (**C**) Flow cytometry analysis of apoptosis in U266 cells transfected with siRNA vectors and then treated by ATRA at indicated concentrations for 72 h. Data are typical of three similar experiments. The percentage of Annexin V-FITC and/or PI positive cells was depicted with cytofluorometer quadrant graphs.

Given that Ape/Ref-1 links with cell proliferation and survival, its upregulation should attenuate the cytotoxicity of ATRA. Next, we examined whether Ape/Ref-1 inhibition enhanced the growth arrest and apoptosis exerted by ATRA. As shown in **Fig. 2A**, specific Ape/Ref-1 siRNA reduced the Ape/Ref-1 protein level by about 70% relative to the control groups. In parallel to Ape/Ref-1 knockdown, U266 cells demonstrated a significant decrease in cellular proliferation. Additionally, Ape/Ref-1 depletion exerted an additive effect with ATRA administration to induce the growth arrest of U266 cells (**Fig. 2B**). Importantly, Ape/Ref-1 depletion greatly facilitated ATRA-induced cell apoptosis (**Fig. 2C**). 10 µM of ATRA caused an 18.3±1.4% apoptosis in U266 cells transfected with vector control at 72 h, but induced a 31.4±2.8% apoptosis in U266 cells expressing Ape/Ref-1-specific siRNA (*P*<0.01). Therefore, upregulated Ape/Ref-1 may alleviate the sensitivity of ATRA treatment.

### ATRA Upregulates Ape/Ref-1 level via p38-MSK Signaling Cascade

**Figure 3:**
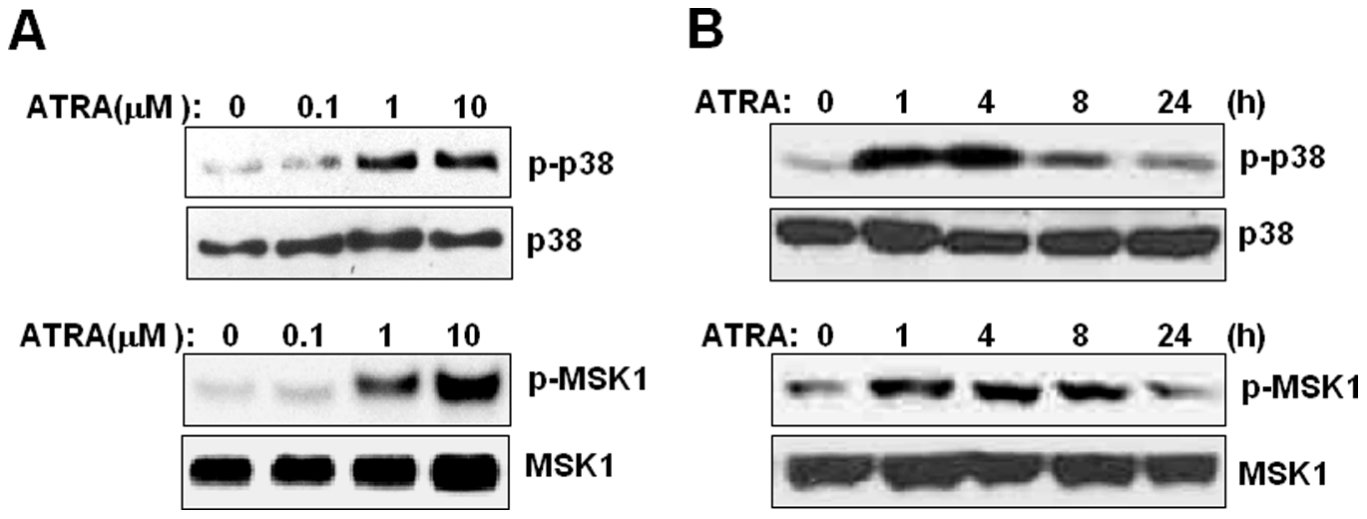
Effect of ATRA on p38-MSK cascade activation in myeloma cells. (**A**) U266 cells were treated with the indicated concentrations of ATRA for 2 h. Cell lysates were subjected to immunoblotting with antibodies to phosphorylated and total p38 as well as MSK1 proteins. (**B**) U266 cells were incubated with 1 µM of ATRA for the indicated periods. Phosphorylated p38 and MSK1 were measured by Western blotting. The experiment was repeated at least three times. The figure shows a representative result.

ATRA usually exerts its effect through the conventional retinoic acid receptor (RAR) to activate its target genes. However, promoter analysis suggest that there is no typical retinoic acid response elements (RARE) located in the proximal promoter of Ape/Ref-1. It has been reported that noncanonical actions of ATRA including the activation of mitogen-activated protein kinase (MAPK) pathways in a variety of cancer cells [29], [30]. In this study, we found that ATRA treatment activated p38 and MSK (mitogen- and stress-activated protein kinase) kinases in a dose-dependent fashion (**Fig. 3A**). MSK kinase lying downstream of MAPK-p38 cascade, can integrate mitogenic signals, pro-inflammatory cytokines, and cellular stress. After treatment with 1 µM of ATRA, the phosphorylation of p38 and MSK kinases in U266 cells increased, to the maximum at 4 h, and remained elevated until 24 hr (**Fig. 3B**).

**Figure 4:**
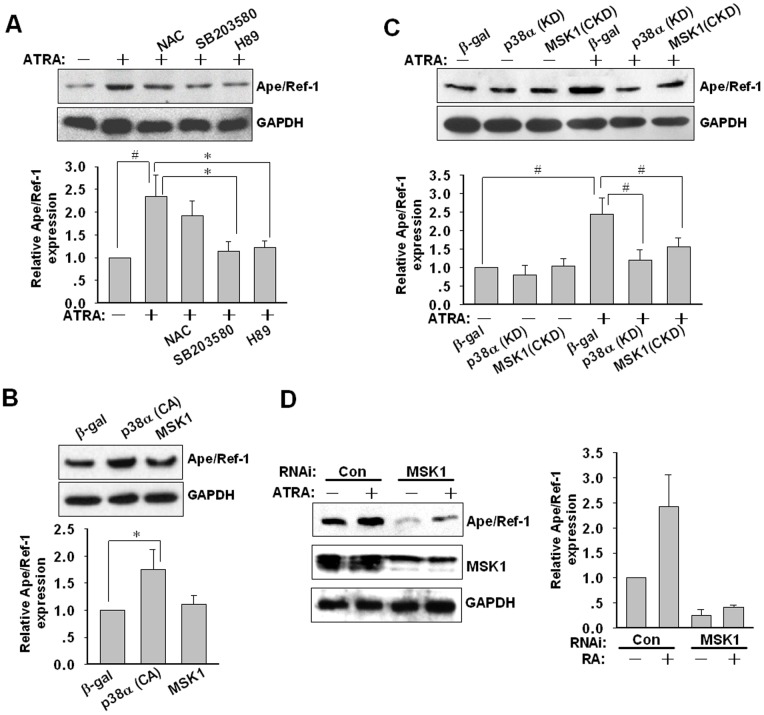
Essential role of p38-MSK activation in Ape/Ref-1 induction. (**A**) U266 cells were incubated with various signal transduction inhibitors (1 mM NAC, 10 µM SB203580, 10 µM H89) in the presence and absence of 1 µM of ATRA for 48 h. Medium and inhibitors were replaced every 24 h. Ape/Ref-1 and GAPDH proteins were detected by Western blotting and semi-quantitative densitometric analysis was performed from three independent experiments. (**B**) After transfection with β-gal, p38α(CA), and MSK1 plasmids for 48 h, U266 cells were lysed for Western blotting followed by densitometric scanning to quantify the relative expression of Ape/Ref-1 and GAPDH proteins. The experiment was repeated at least three times. The figure shows a representative result. (**C**) U266 cells were transiently transfected with expression constructs for β-gal, p38α(KD), and MSK1(CKD). At 24 h after transfection, cells were incubated with 1 µM ATRA for another 48 h. Cell lysates were assayed for Ape/Ref-1 and GAPDH expression. All results represent at least three independent experiments. (**D**) U266 cells were transfected with 100 nM of control or *MSK1* siRNA for 24 h, and then incubated with 1 µM ATRA for another 48 h. Ape/Ref-1 and GAPDH levels were measured and shown in representative blots. The relative levels of Ape/Ref-1 to GAPDH in each group from A was quantified by densitometric analysis and normalized to that of the control group. Data are mean ± SD from three independent experiments. *P<0.05, ^#^ *P*<0.01.

Based on the above data, we hypothesized that p38-MSK cascade would be implicated in the ATRA-induced upregulation of Ape/Ref-1 protein. To verify this point, effects of p38 and MSK inhibition on the expression of Ape/Ref-1 in the presence of 1 µM of ATRA was firstly investigated. The p38 inhibitor SB203580 and the MSK inhibitor H89 significantly attenuated the augment of Ape/Ref-1 expression induced by ATRA (**Fig. 4A**). To investigate the possible impact of oxidative stress on Ape/Ref-1 expression a ROS (reactive oxygen species) scavenger, NAC was used. However, NAC slightly abrogated the ATRA-mediated Ape/Ref-1 augment without significant difference. It indicated that the inducible expression of Ape/Ref-1 by ATRA was irrelevant to oxidative stress. Secondly, transient transfection with a vector encoding a constitutively active p38α (CA) prominently enhanced the Ape/Ref-1 level (*P*<0.05) (**Fig. 4B**). Compared with p38α (CA), forced expression of wild-type MSK1 exhibited a much less efficiency in Ape/Ref-1 expression (**Fig. 4B**). In spite of this, overexpression of kinase-defective p38α (KD) and MSK1 (CKD) both aborted the increased expression of Ape/Ref-1 induced by ATRA (**Fig. 4C**).

To further confirm the role of MSK in the Ape/Ref-1 upregulation by ATRA, we knockdown the MSK1 level using specific *MSK1* siRNA. As shown in **Fig. 4D**, silence of *MSK1* using siRNA reduced the constitutive expression of Ape/Ref-1, and remarkably blocked its inducible expression by ATRA. This finding strengthened the important role of MSK in Ape/Ref-1 expression. Taken together, these results implicate that the inducible expression of Ape/Ref-1 mainly attributes to p38-MSK activation by ATRA stimulation.

### CREB Phosphorylation by MSK Upregulates Ape/Ref-1 Expression

**Figure 5:**
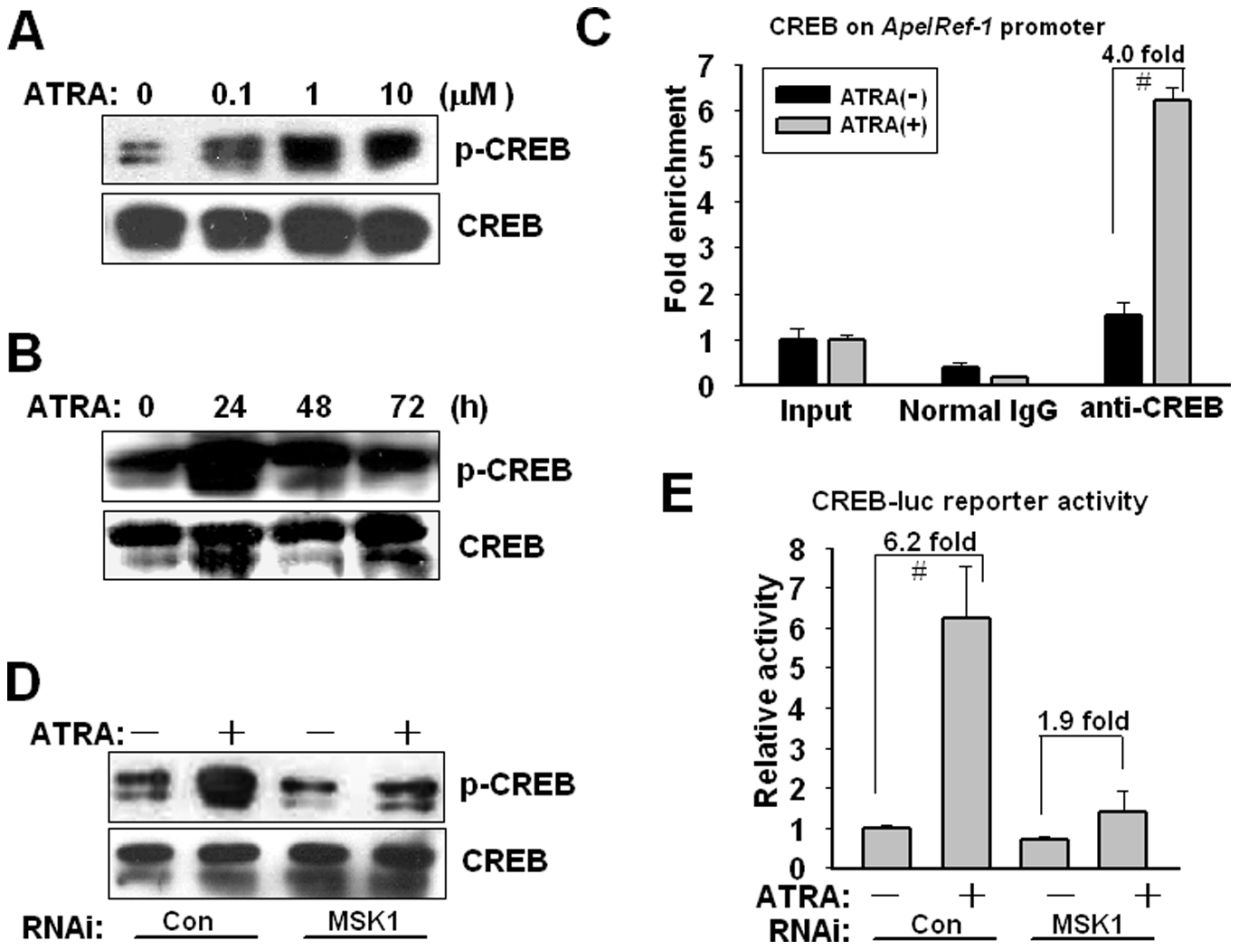
MSK1-mediated CREB phosphorylation activates Ape/Ref-1 expression. (**A**) U266 cells were treated with the indicated concentrations of ATRA for 4 h. Cell lysates were subjected to immunoblotting with antibodies to phosphorylated CREB (Ser133) and total CREB. (**B**) U266 cells were incubated with 1 µM of ATRA for the indicated periods. Phosphorylated and total CREB levels were measured by Western blotting. The figure shows a representative result of triplicate experiments. (**C**) After exposure to 1 µM ATRA for 24 h, U266 cells were subjected to ChIP experiments using anti-CREB and control normal IgG antibodies. Immunoprecipitated genomic DNA fragments were amplified by PCR with specific primers targeting *Ape*/*Ref-1* promoter. Input reflected the relative amounts of sonicated DNA fragments using in immunoprecipitation. ATRA vs. vehicle: ^#^ *P*<0.01, n = 3. (**D**) At 24 h after transfection of scrambled or *MSK1* siRNAs, cells was treated with 1 µM of ATRA for another 24 h. Attenuated CREB phosphorylation by *MSK1* knockdown was detected by Western blotting. (**E**) At 24 h after transfection of siRNAs against *MSK1* together with CREB-luc (2 µg) and pRSV-luc (20 ng) reporters, U266 cells were incubated with 1 µM ATRA for another 24 h. Luciferase activity was measured and normalized to *Renilla* luciferase activity. Each value presented is the average of triplicate samples and a representative of multiple independent experiments.

Activated MSK is known to phosphorylate and activate various transcription factors, such as CREB, SRF, and NF-κB. CREB is implicated in ATRA-mediated cell differentiation, and is a crucial transcriptional factor regulating the expression of *Ape*/*Ref-1* [30]–[33]. In this study, ATRA treatment for 4 h was coupled with an induced phosphorylation of CREB at Ser133 in a dose-dependent manner (**Fig. 5A**). As shown in **Fig. 5B**, CREB activation reached a peak after 1 µM of ATRA treatment for 24 h, and then declined thereafter. Quantitative ChIP PCR showed that the recruitment of CREB onto *Ape*/*Ref-1* promoter was augmented by 4.0-fold after 1 µM of ATRA treatment for 24 h (**Fig. 5C**). Furthermore, *MSK1* RNAi abrogated CREB phosphorylation induced by ATRA, indicating MSK1 is a necessary requisite for this phosphorylation (**Fig. 5D**). U266 cells were transfected with CREB-luc plasmid containing four repeated CREB binding sites and relative luciferase activity was measured after 1 µM of ATRA stimulation for 24 h. ATRA increased the reporter activity by 6.2-fold, but this increased acivity was greatly weakened by *MSK1* RNAi (**Fig. 5E**). These results suggest that MSK1-mediated CREB phosphorylation is required for Ape/Ref-1 upregulation induced by ATRA.

### CBP Upregulation Facilitates Ape/Ref-1 Expression

**Figure 6:**
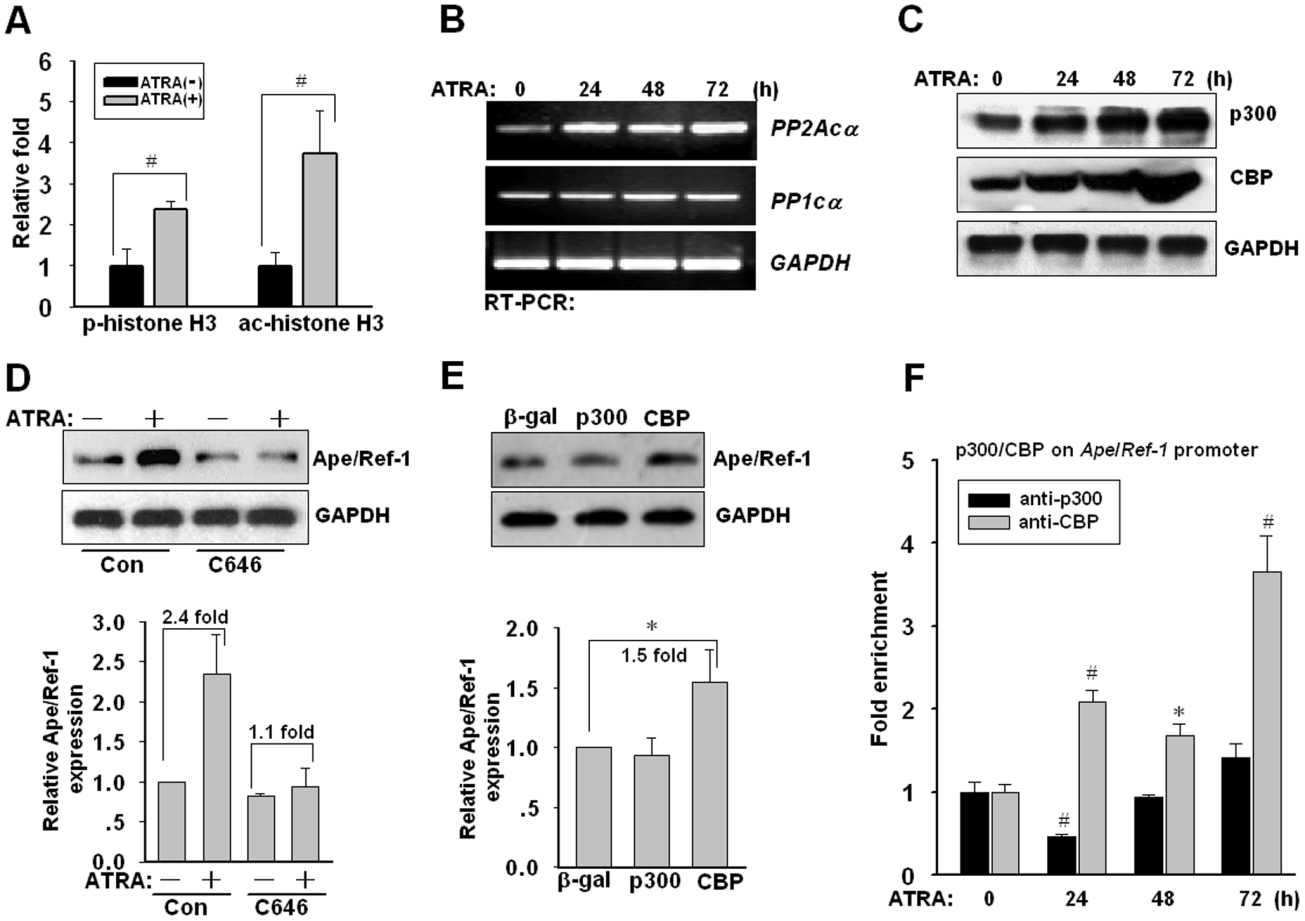
CBP induced by ATRA contributes to Ape/Ref-1 expression. (**A**) U266 cells were treated with 1 µM of ATRA for 4 h and subsequently ChIP experiments were performed with anti-phosphorylated (Ser10) or acetylated (Lys9) histone H3 antibodies. (**B**) After 1 µM ATRA treatment for different periods, RT-PCR was performed to analyze the expression of *PP2Acα* and *PP1cα*. *GAPDH* was used as an internal control. Each assay was performed in triplicate. The figure shows a representative result of triplicate experiments. (**C**) U266 cells were incubated with 1 µM ATRA for the indicated periods. CBP and p300 expression were measured by Western blotting. (**D**) U266 cells was pretreated by 20 µM of C646 for 1 h, and then incubated with ATRA for 48 h. The medium and additives were replaced every 24 h. Western blotting was performed to detect Ape/Ref-1 expression. (**E**) U266 cells were transfected with p300 or CBP expression vectors. 48 h later after transfection, the cell lysate was subjected to Western blotting using the anti-Ape/Ref-1 antibody. (**F**) U266 cells were treated with 1 µM ATRA for dedicated periods and subsequently ChIP experiments were performed in triplicate with anti-p300 or CBP antibodies. *P<0.05, ^#^ *P*<0.01, versus the control group untreated by ATRA, n = 3.

Because the activation of MSK can initiate a rapid alteration of phosphorylation and acetylation status of histone H3, we estimated the phosphorylation level of histone H3. ChIP assays revealed that the phosphorylation (Ser10) and acetylation (Lys9) of histone H3 on the *Ape*/*Ref-1* promoter were increased upon ATRA stimulation for 4 h (**Fig. 6A**). Therefore, chromatin remodeling mediated by p38-MSK cascade links to Ape/Ref-1 expression modulated by ATRA. Nevertheless, there exist several phosphatases governing the dephosphorylation of p38 cascade and histone H3, including protein phosphatase 1 (PP1) and phosphatase type 2A (PP2A) [34], [35]. We also observed a persistent rise in mRNA level of PP2A catalytic subunit α (*PP2Acα*) following ATRA treatment (**Fig. 6B**). In contrast, the mRNA level of PP1 catalytic subunit α (*PP1cα*) displayed less significant changes during the ATRA treatment. Thus, we speculate that p38-MSK activation and histone phosphorylation contribute to the temporary induction of Ape/Ref-1 expression, and the prolonged upregulation could derive from other mechanisms such as histone acetylation, a stable mark of gene activation.

It is well known that CBP and p300, containing histone acetyltransferase (HAT) activity, are required for ATRA-induced differentiation and growth arrest [36]. Hence, we examined the expression of CBP and p300 after ATRA treatment. As shown in **Fig. 6C**, ATRA treatment augmented both the CBP and the p300 protein levels. C646, a selective pharmacological inhibitor of CBP/p300 activity, partly alleviated the basic expression of Ape/Ref-1 and completely antagonized the ATRA-induced Ape/Ref-1 upregulation (**Fig. 6D**). Intriguingly, transient transfection assays indicated that CBP, but not p300, prominently enhanced the expression of Ape/Ref-1 in the absence of ATRA (*P*<0.01) (**Fig. 6E**). This result preliminarily demonstrates that CBP and p300 play very distinct roles in *Ape/Ref-1* gene transcription. Concomitant with progressive augment of CBP protein after ATRA treatment, the enrichment of CBP on the *Ape*/*Ref-1* promoter also accordingly enhanced (**Fig. 6F**). Conversely, p300 on the *Ape*/*Ref-1* promoter firstly underwent a decline at 24 h and then gradually elevated. Thus, an adequate amount of CBP would function as a cofactor in association with CREB and stably establish a transcriptional active status on the *Ape*/*Ref-1* promoter.

### ATRA Confers Chemoresistance to Melphalan

**Figure 7:**
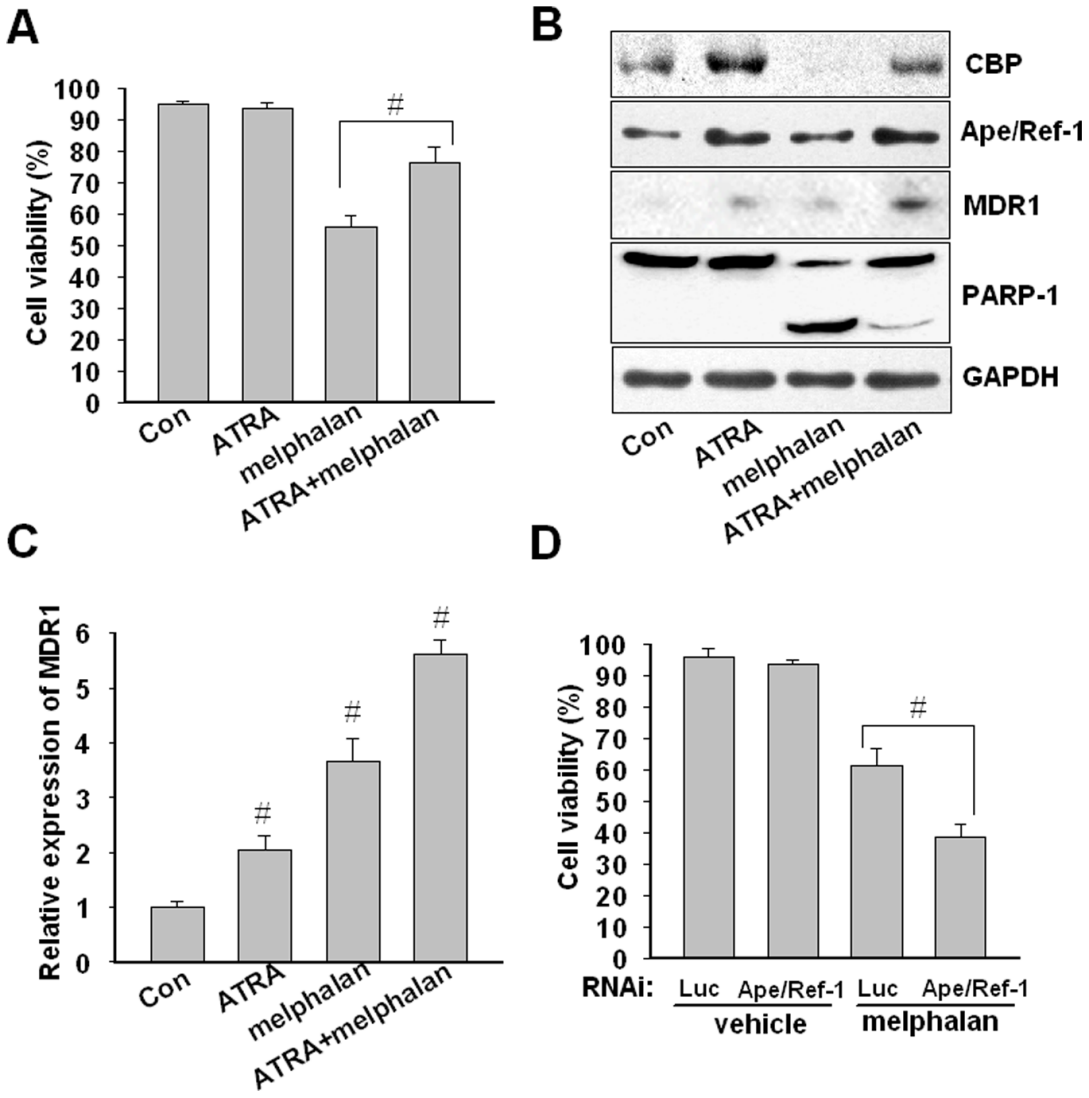
ATRA pretreatment inhibits melphalan-induced apoptosis. (**A**) U266 cells were treated with 1 µM of ATRA or vehicle for 72 h prior to 5 µM melphalan treatment for another 48 h. Apoptosis in cells treated or untreated by melphalan was analyzed by flow cytometry. The percentage of survival cells in each group was calculated from triplicate data. Data are mean ± SD from three independent experiments.^ #^ *P*<0.01 by one-way ANOVA. (**B**) U266 cells treated as described above were subjected to immunoblotting with antibodies to CBP, Ape/Ref-1, MDR1, PARP-1, and GAPDH proteins. The experiment was repeated at least three times. The figure shows a representative result. (**C**) The relative expression of *MDR1* was determined by quantitative real-time PCR and normalized to *GAPDH* mRNA level. All experiments were performed in triplicate, and data are shown as normalized mean ± SD. ^#^ *P*<0.01, n = 3. (**D**) After transfected with siRNA or control vectors for 24 h, U266 cells were treated with melphalan for 48 h and cell apoptosis was monitored by flow cytometry analysis.

Ape/Ref-1 is associated with tumor cell resistance to various chemotherapeutic agents. Next, we examined the possible contribution of ATRA pretreatment to the chemoresistance response in myeloma cells. Pretreatment with 1 µM of ATRA for 72 h prior to exposure to 5 µM of melphalan (an alkylating chemotherapeutic agent) for another 48 h, the apoptosis of U266 cells was further quantified with flow cytometry by double-staining of Annexin V-FITC and PI. As shown in **Fig. 7A**, U266 cells pretreated by ATRA developed resistance to melphalan marked by a significantly decrease in mortality percentage from 76.3±5.0% to 56.1±3.3% (*P*<0.01). In addition, melphalan-induced PARP-1 cleavage as a hallmark of apoptosis was significantly depressed by ATRA pretreatment (**Fig. 7B**). In accord with the above findings, ATRA pretreatment increased the expression of Ape/Ref-1 and CBP (**Fig. 7B**). Prominently, ATRA reversed the melphalan-induced downregulation of CBP protein (**Fig. 7B**). Moreover, ATRA pretreatment facilitated the expression of MDR1, a known downstream target of Ape/Ref-1 (**Fig. 7B and C**). And the sequential combination of ATRA and melphalan further elevated MDR1 expression (**Fig. 7B and C**). On the other hand, we also observed that Ape/Ref-1 blockade by RNAi can promote the pro-apoptotic activity of melphalan (**Fig. 7D**). Obviously, upregulated Ape/Ref-1 by ATRA is adverse to melphalan-mediated cytotoxicity. Taken together, our results indicate that ATRA treatment can render resistance to anticancer drugs via a mechanism of Ape/Ref-1.

## Discussion

ATRA, the most abundant natural retinoid, is widely studied in clinical trials of multiple myeloma due to its potent differentiation-promoting, pro-apoptotic, and growth-suppressive effects. However, one intractable obstacle for ATRA therapy is the emergence of intrinsic and acquired resistance. The intrinsic resistance to ATRA mostly attributes to genetic or epigenetic aberrations. For instance, ATRA-resistant HL-60 and NB4 cells were shown to contain mutations in the ligand binding domain of RARα [1]. Particularly, the occurrence of secondary, acquired resistance is more common in clinical therapies continuously with ATRA as a single treatment. Single treatment with ATRA can induce the expression of several pro-survival or multidrug resistance proteins, or affect their function [1]. In this study, we found that ATRA enhances chemoresistance of myeloma cells via upregulating Ape/Ref-1 in a noncanonical manner.

Our results indicated that a suitable dose range of ATRA treatment effectively led to an increase in Ape/Ref-1 protein in myeloma cells. The optimal dosage of ATRA at 1 µM caused the maximal increase in Ape/Ref-1 expression. Upregulated Ape/Ref-1 probably contributes to cell survival from various deleterious agents including ATRA and other chemotherapeutic drugs. In fact, our study also showed that ATRA at low dosage preferred to induce growth arrest rather than apoptosis, on the contrary, high dosage of ATRA can trigger a significant apoptosis in U266 and RPMI8226 cells. Therefore, different dosage of ATRA exhibits different effect on myeloma cells which are sensitive to ATRA treatment. In this study, we found ATRA treatment prior to melphalan indeed facilitated cell escape from apoptosis. Ape/Ref-1 is a multifunctional protein involved in the maintenance of genomic integrity and in the regulation of gene expression [23], [24]. Primarily, Ape/Ref-1, as an essential enzyme in the base excision repair pathway for the repair of DNA damage, since melphalan is an alkylating chemotherapeutic agent used for cancer therapy in clinic [37], and Ape/Ref-1 is a well-known important factor for repairing DNA damage induced by alkylating agents [23], therefore, we established a convincing link between Ape/Ref-1 and drug resistance in myeloma cells. Besides, Ape/Ref-1 maintains numerous transcription factors in an active reduced state and stimulating their DNA binding activities. Due to diverse functions, Ape/Ref-1 influences multiple tumor survival mechanisms, including growth, proliferation, metastasis, angiogenesis, and stress responses. For instance, hypoxic stress in the tumor microenvironment evokes Ape/Ref-1 to facilitate the DNA binding activity of HIF-1α, triggering the expression of downstream genes including vascular endothelial growth factor (VEGF), miR210, and CA-9 [38]. Ape/Ref-1 is also a vital cofactor of NF-κB and forms a transcription machine to regulate *Bcl-2* expression [39]. Therefore, Ape/Ref-1 promotion might directly abrogate the pro-apoptotic effects of ATRA and melphalan. Importantly, Ape/Ref-1 overexpression causes a decrease in the bioavailability of ATRA and other chemotherapeutic drugs. Because Ape/Ref-1 stably interacts with YB-1 and enhances its binding to the Y-box element, activating the expression of *MDR1* gene [21], [22]. Previous report revealed that ribozymes targeting MDR1 rendered increased retinoid sensitivity in the ATRA-resistant HL60 cells [19]. Thus, Ape/Ref-1 upregulation is relevant with the acquisition of MDR1-mediated multidrug resistance. Taken together, ATRA-mediated Ape/Ref-1 upregulation protects cell from apoptosis via multiple mechanisms.

Promoter analysis revealed CREB and ATF4/c-Jun binding sites on the Ape/Ref-1 promoter are responsible for its inducible expression after toxic exposures [24], [32]. In this study, we observed ATRA-induced CREB activation concomitantly with a significant increase of CREB on the *Ape/Ref-1* promoter. CREB is an important transcriptional factor regulating the expression of several anti-apoptotic genes, including *Bcl-2* and *Survivin* [40]. CREB activation is required for the differentiation-promoting effects of noncanonical ATRA signaling pathway which is independent on conventional retinoic acid receptors [30], [31], [41], [42]. Herein, our findings also support CREB activation by ATRA stimulation constitutes a pro-survival signal via upregulating Ape/Ref-1. Rapid activation of CREB by ATRA treatment should yield binary roles in cell differentiation and resistance to stress conditions.

In this study, we further discovered the involvement of p38-MSK cascade in regulation of Ape/Ref-1 expression. MSK mediates signal transduction downstream of p38 and ERK kinases, integrating mitogenic and cellular stress signals. Moreover, MSK targets CREB and histone H3 for phosphorylation with far greater efficacy than p90 ribosomal S6 kinase (RSK), which is only activated by ERK cascade [34], [35]. Herein, we found depletion of MSK1 via siRNA or overexpression of dominant-defective MSK1 greatly abolished ATRA-mediated Ape/Ref-1 expression. However, we also observed that sustaining treatment of ATRA simultaneously induced the upregulation of phosphatase PP2A, constituting a negative feedback loop to limit the activation of p38 cascade. Therefore, rapid activation of p38-MSK-CREB cascade accounted for the early induction of Ape/Ref-1 upon ATRA exposure, but persisted upregulation of Ape/Ref-1 probably originated from other mechanisms.

CBP and p300 function as transcriptional cofactors and histone acetyltransferases, and are integrators of many signaling pathways that govern transcription. CBP and p300 share similar protein interaction domains binding to CREB, and modulate target genes downstream of CREB. In this study, we also found ATRA treatment promoted induction of CBP/p300 in myeloma cells, which contributes to CREB acetylation and in turn regulates the Ape/Ref-1 expression. CBP/p300-mediated chromatin remodeling maintains a transcriptional active status of inducible target genes. Interestingly, overexpression of CBP, rather than p300, directly led to an augment in Ape/Ref-1 expression. CBP recruitment on the *Ape/Ref-1* promoter, but not p300, significantly enhanced after ATRA treatment. Although CBP and p300 are highly homologous and share similar functional properties, there have individual activities in modulating Ape/Ref-1 expression. Other evidences indicated that CBP, but not p300, is crucial for hematopoietic stem cell self-renewal and *survivin* gene expression [43], [44]. In contrast, p300, but not CBP, is essential for proper hematopoietic differentiation [43]. We assumed that the distinct role of CBP and p300 in Ape/Ref-1 regulation may attribute to the difference in modulating CREB. CREB-CBP interaction is more limiting or critical than the CREB-p300 interaction for several biological events [45]. For instance, CREB knock-out mice exhibited a learning deficit similar to that of CBP mutant mice, but not mice harboring mutant p300.

In summary, our results demonstrated a potential novel mechanism that ATRA induces chemoresistance via upregulating Ape/Ref-1 expression in a noncanonical manner. The discovery of inhibitors directing the DNA repair and redox activities of Ape/Ref-1 is underway. The utilization of ATRA in combination with Ape/Ref-1 inhibitors may yield a comprehensive effect in tumor growth suppression and remission of chemoresistance.
